# Fusobacterium nucleatum: a new player in regulation of cancer development and therapeutic response

**DOI:** 10.20517/cdr.2021.144

**Published:** 2022-05-12

**Authors:** Tengda Zhao, Xueping Wang, Liwu Fu, Ke Yang

**Affiliations:** ^1^Department of Oral and Maxillofacial Surgery, Department of Health Management Center, Shandong Provincial Hospital Affiliated to Shandong First Medical University, Jinan 250021, Shandong, China.; ^2^Sun Yat-sen University Cancer center, State Key Laboratory of Oncology in Southern China, Collaborative Innovation Center for Cancer Medicine, Guangzhou 510060, Guangdong, China.

**Keywords:** *Fusobacterium nucleatum*, tumor microenvironment, immune evasion, metastasis, chemoresistance

## Abstract

A dysbiosis in microbial diversity or functionality can promote disease development. Emerging preclinical and clinical evidence emphasizes the interplay between microbiota and both disease evolution and the treatment response of different cancers. One bacterium that has garnered much attention in a few cancer microbiota studies is *Fusobacterium nucleaum* (Fn). To provide updated knowledge of the functional role of Fn in cancer prevention and management, this review summarizes the relationship among Fn, cancer, and chemoimmunotherapy response, with the potential mechanisms of action also intensively discussed, which will benefit the development of strategies to prevent or treat cancer via Fn-based therapeutic interventions.

## INTRODUCTION

Cancer, as the second leading disease-related cause of death in humans worldwide, affects almost all body regions^[1]^. Currently, cancer progression and resistance to therapy remain major challenges in cancer treatment and the main causes of poor prognosis^[2]^. Among the factors that contribute to cancer development and chemotherapy response, in addition to host genetic susceptibility and environmental exposures, a new and important player is emerging in regulating the development and drug resistance of cancer: microbiota^[3]^.

The conventional paradigm proposes that a balanced microbiota is positively health-associated, while damage in microbial diversity or functionality, including dysbiosis or unbalanced microbiota, can promote the development of disease, such as various cancers^[3-5]^. Emerging preclinical and clinical evidence links the microbiota and their metabolites with carcinogenesis^[6]^. Related studies revealed that cancer formation can be driven by microbial pathogens in 15%-20% of cancer cases^[7]^. Bacteria *Prevotella gingivalis*, *Helicobacter pylori*, *Salmonella typhi*, *Prevotella melaninogenica*, *Chlamydia pneumoniae*, *Streptococcus mitis*, *Streptococcus bovis*, and *Capnocytophaga gingivalis* may cause different types of cancers in humans^[8-12]^. Importantly, recent insights shed light on the influence of the microbiota on the response to chemotherapy. The interplay between the microbiota and both tumor evolution and the treatment response of different cancers, especially colorectal cancer (CRC), has recently been studied^[13]^. One bacterium that has garnered wide interest in a few cancer-related microbiota studies is *Fusobacterium nucleatum* (Fn). Although Fn has been considered as an opportunistic pathogen for infections, its role as a cancer- or chemoresistance-causing member is revealed in various ways, by which Fn contributes to cancer initiation, progression, and the response to chemotherapy.

Based on these findings, we undertook a systematic review of the role of Fn as an oncobacterium. The research included in this review covers a period of 20 years, until the end of November 2021.

## THE ROLE OF THE Fn IN CARCINOGENESIS

**Figure 1:**
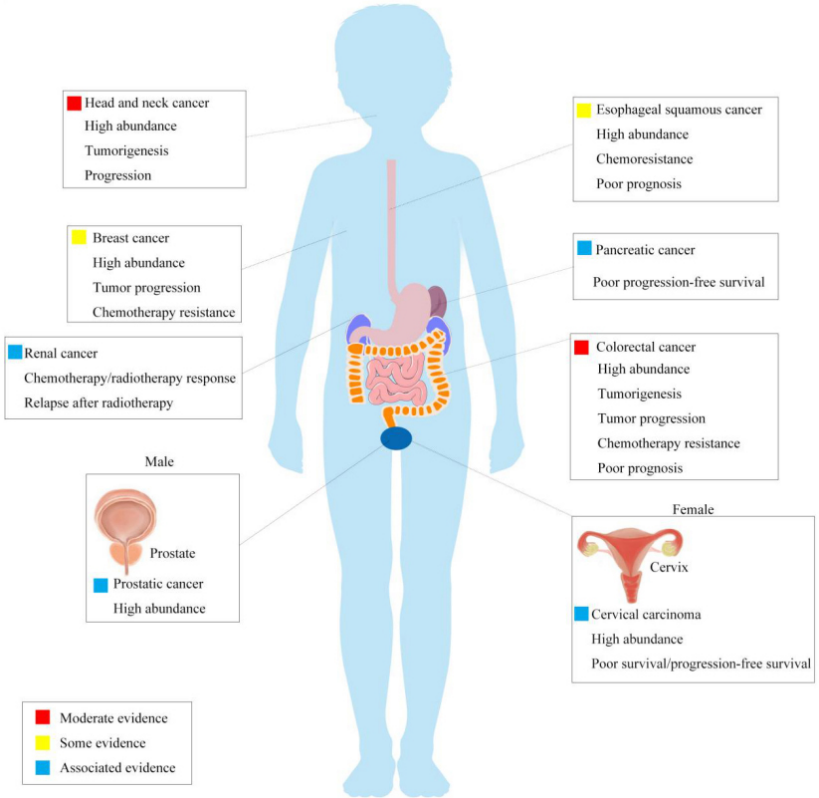
Different cancers associated with Fn. Fn is a Gram-negative anaerobic bacillus that exists, among others, in the human oral cavity and the gastrointestinal tract. For carcinogenesis, the high abundance of Fn is associated with head and neck cancer, esophageal cancer, pancreatic cancer, prostatic cancer, cervical carcinoma, and breast cancer. Relative events reported in each cancer are listed in the rectangles. Fn: *Fusobacterium nucleaum*.

Fn is a Gram-negative anaerobic bacillus that exists, among others, in the human oral cavity and the gastrointestinal tract. It exerts pro- or anti-pathogenic effects in the oral cavity affecting human periodontal health and diseases. The high abundance of Fn has been reported to be associated with head and neck cancer, esophageal cancer, pancreatic cancer, prostatic cancer, cervical carcinoma, and breast cancer [Figure 1]^[14-18]^. Some investigators proposed that Fn is a passenger that multiplies under favorable conditions induced by malignant tumors^[19]^. However, more research data support Fn as having a mechanistic role in driving carcinogenesis rather than being a microbial passenger, whose effects can be classified into three steps: initiation, promotion, and progression^[20]^. In the following section, we review studies on the mechanisms by which Fn initiates and promotes carcinogenesis and enhances disease progression.

### The first step: adhering to and invading human epithelial and endothelial cells

#### Fn accumulates in distant organ

Adherence and invasion are essential mechanisms for oncobacterium colonization, dissemination, and evasion, subsequently inducing a series of host responses. As one of the most abundant bacteria in the oral cavity, Fn can cause infectious inflammatory conditions at multiple body sites in addition to inflammation of the gingival tissue^[21-24]^. It has also been proved that mislocalization of Fn is associated with many cancers. Although it seems possible that Fn directly spreads from the oral cavity to the colon, certain evidence demonstrates that Fn reaches distant sites of inflammation and tumorigenesis via a hematogenous route^[25,26]^. However, how can an oral bacterium be implicated in so many infections and cancers within and outside the mouth? The main answer lies in two key virulence proteins expressed by Fn, FadA and Fap2, which are responsible for localization and colonization^[27-30]^.

#### Mechanisms of the key virulence proteins: FadA and Fap2

FadA of Fn has previously been identified to bind host cells. FadA mainly exists in two forms: the intact pre-FadA anchored to the membrane, comprising 129 amino acids, and the secreted mature FadA (mFadA), consisting of 111 amino acids^[31]^. Pre-FadA and mFadA elicit internalization and form an active complex, FadAc, which leads to binding to host cell-junction molecules, cadherins^[31]^.

Two host receptors, epithelial cadherin (E-cadherin) and vascular endothelial cadherin (VE-cadherin), are found on host epithelial and endothelial cells, respectively^[32,33]^. As cadherins are widely expressed in various tissues and cells, the binding of FadA to cadherins likely explains why it can colonize different sites outside oral. Further, the binding of FadAc and VE-cadherin on endothelial cells promotes VE-cadherin to transfer from cell-cell junctions to intracellular compartments, which increases the permeability of the endothelium.

**Figure 2:**
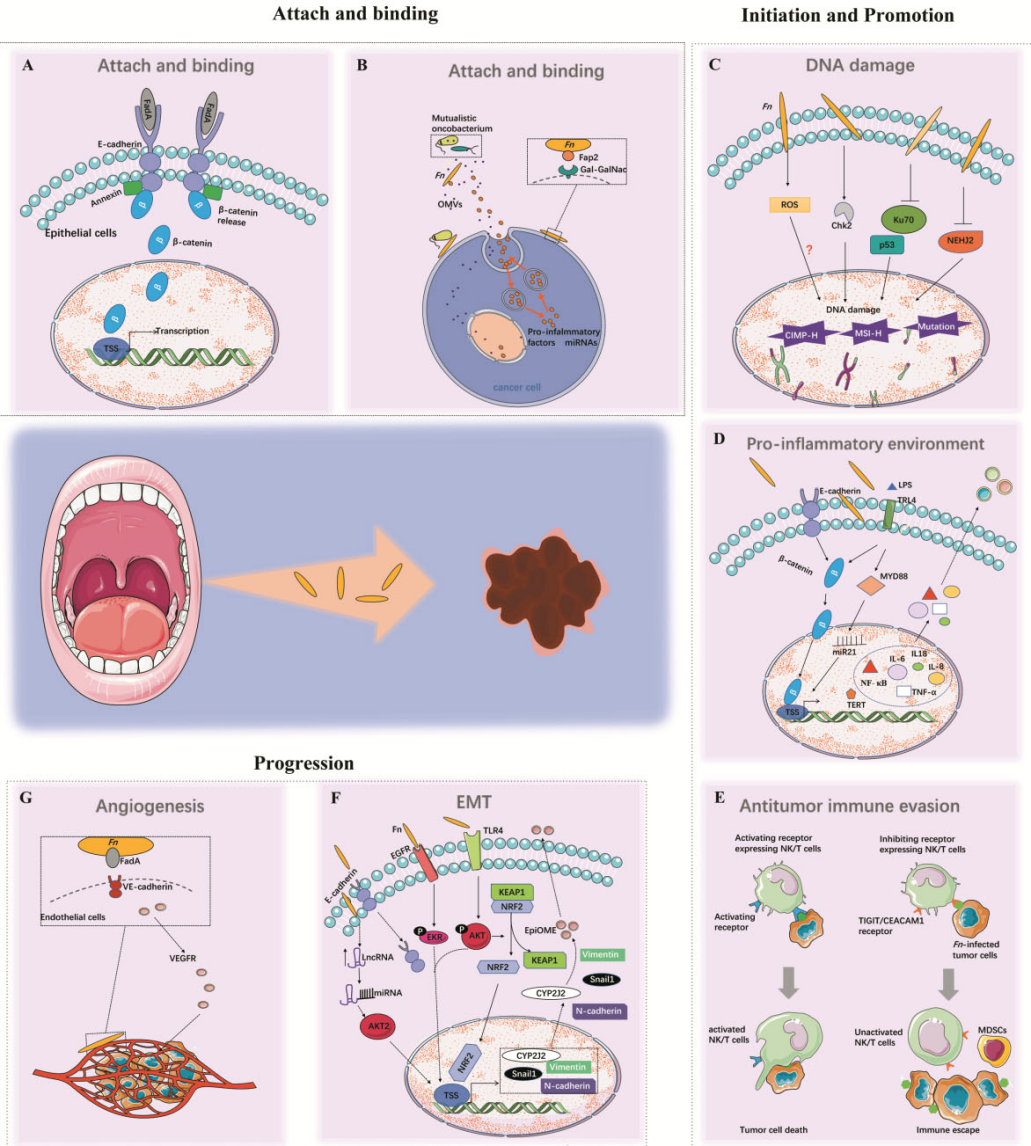
The mechanisms by which Fn initiates and promotes carcinogenesis and enhances disease progression. Fn plays an important role in the whole process of cancer carcinogenesis and progression, supporting the notion that Fn may have a causative role in different states of cancer rather than being a consequence of cancer development. (A,B) Mechanisms of FadA and Fap2 on host cell adherence and invasion. (C-E) Host responses for cancer initiation and promotion including DNA damage, antitumor immune evasion, and pro-inflammatory environment. (F,G) Fn-induced tumor invasion and metastasis for cancer progression. Fn: *Fusobacterium nucleaum*.

After the activated FadAc binds specifically to the transmembrane domains of E-cadherin or VE-cadherin^[34]^, the molecules form a FadA-E-cadherin-AnnexinA1 (ANXA1)-β-catenin complex, which is then internalized to induce a series host responses, such as activation of β-catenin signaling and elevated levels of oncogenic or inflammatory genes, transcription factors, and Wnt-related genes, thereby contributing to cancer initiation and promotion [Figure 2A]^[35]^.

The other Fn protein, the lectin Fap2, is an autotransporter protein. A transposon screen revealed the significance of Fap2 in mediating bacteria enrichment by binding of microbial and host cells^[36]^. An overexpressed D-galactose-b (1-3)-N-acetyl-D-galactosamine (Gal-GalNAc) can be recognized by fusobacterial Fap2. Then, Fap2 directly binds to Gal-GalNAc and functions as a Gal-Gal-NAc lectin to mediate Fn attachment to tumor epithelial cells^[37]^, which subsequently inhibit immune cell cytotoxicity and activity [Figure 2B]^[38]^.

### The second step: inducing host response for tumor initiation and promotion

#### DNA damage

DNA damage is broadly acknowledged to facilitate tumor initiation and promotion^[39]^. It is recognized that bacteria could directly enhance DNA methyltransferase activity in cell lines and animal models^[40,41]^. Fn infection is pervasive and is associated with tumor suppressor gene (TSG) promoter methylation^[40]^, promoting high microsatellite instability (MSI-H) and a high level of CpG island methylation (CIMP-H)^[42]^. Kelly *et al.*^[43]^, Mima *et al.*^[44,45] ^and Ito *et al.*^[46]^ discovered that a high load of Fn was positively correlated with CIMP, MSI-H, and BRAF mutation in CRC tissues by univariate analysis. Tahara *et al.*^[47] ^also found that Fn-high CRCs were enriched in CIMP, MSI-H, hMLH1 methylation, wild-type p53, and mutant CDH7/8. Mechanistically, first, Fn might trigger TSG promoter hypermethylation by regulating the DNA methyltransferase. Interestingly, Lee *et al.*^[48] ^also revealed that Fn of high-load patients had higher rates of transition mutation and nucleotide change of C to T (G to A) compared with Fn-low patients regardless of MSI status. Additionally, Fn-high tumors were positively correlated with a higher mutation rate of APC membrane recruitment 1 and Ataxia telangiectasia mutated genes^[48]^. Thus, Fn may induce promoter DNA methylation or genic mutation to drive tumorigenesis; however, the mechanism by which Fn affects these epigenetic or genetic alterations is not well understood. One potential interpretation is that Fn, involved in inflammation signals, enhances the production of reactive oxygen species (ROS) and recruits inflammatory cytokines. Although many reports suggest that ROS is associated with DNA hypermethylation, currently, there is no obvious evidence to prove that oxidative DNA damage causes genome-wide hypermethylation of promoter CpG islands and CG sites at other parts of the genome^[42]^. One other possible mechanism for gene alteration is the dysregulation of Checkpoint kinase 2 (Chk2), which induces cell cycle arrest and apoptosis upon DNA damage. The data imply that Chk2 is related to DNA damage and progression via Fn-induced E-cadherin/β-catenin pathway activation^[49]^.

Once DNA damage occurs, DNA double-strand breaks (DSBs), the most serious type of DNA damage, are repaired by homologous recombination and nonhomologous end joining (NHEJ). However, a deficient repair process results in malignant transformation. Ku70, an NHEJ initiation molecule, participates in DNA damage repair signaling by initiating apoptosis programs and activating cell cycle detection points. Geng *et al.*^[50]^ reported in 2020 that Fn could cause DNA damage and promote cell proliferation via the Ku70/p53 pathway in oral cancer cells. When suffering from an infection, Nei-like DNA glycosylase 2 (NEIL2), an oxidized base-specific DNA glycosylase, significantly relieves inflammation response and DNA damage. However, the protein level of NEIL2 is reported to be reduced in the progression of several types of cancer^[51]^. A recent study demonstrated a suppressed expression of NEIL2 by Fn infection, which consequently increased DSB accumulation and inflammatory responses, contributing to the initiation and progression of CRC^[52]^.

In summary, Fn infection induced promoter DNA methylation, genetic mutations, and a deficient DNA damage repair process, and hence it probably plays an essential role in tumor initiation and development [Figure 2C].

#### Pro-inflammatory microenvironment

Inflammation is well-recognized as a dominant force in cancer initiation. The NF-κB signaling pathway plays a vital role in activating the transcription of many inflammatory genes. Binding of FadAc to the transmembrane domains of E-cadherin induces phosphorylation and internalization of E-cadherin and accumulation of β-catenin, which consequently leads to the activation of β-catenin-regulated transcription (CRT)^[32]^. Alternatively, Fn also activates β-catenin signaling through its lipopolysaccharides (LPS) via a TLR4/PAK1/β-catenin S675 cascade in CRC cells^[53]^. Then, activated CRT increases the expression of Wnt signaling genes (such as wnt7a, wnt7b, and wnt9a), NF-κB (such as NF-κB2), pro-inflammatory cytokines [including interleukin-6 (IL-6), IL-8 and IL-18], tumor necrosis factor-α (TNF-α), cyclooxygenase-2 (Cox-2), transcription factors (such as the lymphoid enhancer factor), T cell factors (such as TCF1, TCF3, and TCF4), and the oncogenes myc and cyclin D1^[32,54]^. In addition, Fn-infected cells could enhance the expression of microRNA-21 (miR21) via activating TLR4 signaling to MYD88, which consequently activates the NF-κB pathway and elevates telomerase reverse transcriptase expression^[55]^. The former is correlated with the inflammatory response, while the latter confers an unlimited replicative potential to initial tumor cells^[56]^. Fn further promotes the release of these inflammatory cytokines, particularly IL-8, IL-10, and TNF-α, and inflammasomes to form a pro-inflammatory microenvironment that accelerates cancer progression^[31,54,57]^. Inflammasomes contain multiprotein complexes including the apoptosis-associated speck-like protein containing a CARD (ASC), procaspase-1, and a sensor protein, which is either a NOD-like receptor or an absent in melanoma 2 (AIM2)-like receptor^[58]^. In oral squamous cell carcinoma (OSCC) cells, Fn infection promotes AIM2 inflammasome expression and potentially upregulates IL-1β expression^[59]^. These collective findings strongly suggest that the Fn-infected tumor microenvironment is highly inflammatory [Figure 2D].

### Immunosuppressive microenvironment

#### NK cell and T cell inhibition

Immune evasion is a fundamental hallmark of cancer. One mechanism by which Fn causes immune evasion of tumor cells is the inhibition of the cytotoxicity and activity of natural killer (NK) cells. As a part of the innate immune system, NK cells directly and indirectly kill viruses, bacteria, cancer cells, and parasites^[60]^. Signals from inhibiting and activating NK cell receptors determine NK cell activity. T-cell immunoglobulin and immunodominant tyrosine-based inhibitory motif domain (TIGIT), an inhibitory receptor expressed on NK cells, T cells, and tumor-infiltrating lymphocytes (TILs), inhibit NK cell and T cell activity or mediate human T cell arrest in the G1 phase of the cell cycle^[61]^. The direct interaction of Fap2 protein of Fn and TIGIT causes inhibition of NK cell cytotoxicity and cytotoxic T lymphocyte cell death [Figure 2E]^[38]^. The amount of Fn is reported to be inversely associated with CD3+ T cell density in CRC tissue^[62]^. Another inhibitory receptor, CEACAM1, a member of the carcinoembryonic antigen-related cell adhesion molecules (CEACAMs), is expressed on the surface of T cells and NK cells^[63,64]^. Previous studies reported that TILs express high levels of CEACAM1 and produce obviously less IFN-γ compared with T cells derived from para-cancer tissue, suggesting a substantial role of CEACAM1 in mediating T cell exhaustion^[65]^. Most recently, Fn was proved to bind to and activate CEACAM1 to inhibit T cell and NK cell activities, which may help tumors evade immune cell attack by an additive mechanism^[65]^. However, the functional mechanism of Fn binding to CEACAM1 is unknown and needs further investigation.

#### MDSC attraction

Another major immunosuppressive cell population is myeloid-derived suppressor cells (MDSCs), a group of CD11b+CD14+CD33+HLADR-immature myeloid cells that express high levels of inducible nitric oxide synthase and arginase-1 and show strong activity in depressing T cell proliferation and inducing T cell apoptosis^[66]^. MDSCs and their effectors are key components of the neoplasm to promote tumor progression^[60,67]^. Cancer-associated Fn selectively attracts MDSCs and increases the myeloid-lineage infiltrating cells including tumor-associated macrophages (TAMs), tumor-associated neutrophils, CD11b+, M2-like TAMs, conventional myeloid dendritic cells (DCs), and CD103+ regulatory DCs [Figure 2E]. These cells were shown to dampen antitumor immunity and promote tumor progression and angiogenesis^[15,66,68-70]^.

### Additional microenvironmental complexities

Fn has evolved in interaction not only with human cells and tissues but also with the oral microbiota. With its long rod shape, Fn could bind with many other microbial cells, such as *Streptococcus sanguinis* (*S. sanguinis*)^[71]^. When co-cultured with *S. sanguinis*, Fn combined with *S. sanguinis*, and together they assembled into highly corncob-like structures; in this way, a single Fn can bind with up to ten *S. sanguinis* cells. This biological behavior of Fn makes it possible to mediate important biofilm-organizing behavior and interactions with host cells [Figure 2B]. In fact, Fn is usually found to be co-existent in tumors with other microorganisms, especially *Leptotrichia spp.* and *Peptostreptococcus spp.*, which mirrors how they are found to be interacting in the oral cavity^[72,73]^. Notably, Fn is frequently found co-occurring with *Campylobacter spp.*, another important gastrointestinal pathogen, in the same cancer tissues^[73]^. Continual intake of *P. gingivalis* and Fn promote tumor progression in a 4-nitroquinoline-1-oxide (4NQO)-induced mouse tongue cancer model by triggering TLR/STAT3 signaling^[74]^. Infection of macrophages by co-culturing with Fn and* P. gingivalis* enhances inflammasome activation more strongly than when infection with Fn alone^[75]^. Fn also strengthens the invasive ability of *P. gingivalis*, indicating that these bacteria may act synergistically to develop an inflammatory-permissive and tumor-promoting environment^[76]^. Together, these observations suggest that crosstalk between the microbiota may function as a contributor to carcinogenesis.

## FINALLY: Fn PROMOTE INVASION AND METASTASIS

Relatively little is known about how Fn might impact cancer progression at the later stages of carcinogenesis and metastasis, which determine poor prognosis in patients, despite the detection of Fn in CRC metastases to the liver and lymph nodes.

### Epithelial-mesenchymal transition

During cancer progression, activation of the epithelial-mesenchymal transition (EMT) program is another complex hallmark, broadly facilitating local invasion and distant metastasis^[77]^. Expression of EMT markers is significantly associated with Fn level in CRC tissues, indicating the potential involvement of Fn in EMT-colitis-associated cancer (CAC) crosstalk during cancer progression^[78]^. The role of Fn in CAC progression was further verified in mouse models, as Fn apparently enhances the aggressiveness and EMT alteration of CRC cells that were treated with dextran sodium sulfate compared with untreated ones. This promoting effect of Fn was dependent on activation of the EGFR/AKT/ERK pathway^[79]^. Previous studies have found that Cytochrome P450 (CYP) monooxygenases, primarily Cytochrome P450 2J2 (CYP2J2), were involved in tumor progression and cancer drug resistance^[80,81]^. 12,13-epoxyoctadecenoic acid (12,13-EpOME), the CYP2J2-mediated metabolite product, was also reported to be associated with various human diseases^[82]^. A recent finding demonstrates that CYP2J2 and its oncogenic metabolite 12,13-EpOME are heavily enriched in Fn-abundance CRC patients. Further, overexpression of CYP2J2 or 12,13-EpOME dramatically promoted the invasion and migration of CRC cells and resulted in a mesenchymal phenotype. Mechanically, Fn infection activates TLR4/AKT/Keap1/NRF2 signaling to upregulate cytochrome CYP2J2 expression in CRC cells, which then increases the production of 12,13-EpOME, finally resulting in EMT^[83]^. Fn drives cell migration by upregulating mesenchymal markers Vimentin, N-cadherin, and snail1 in human noncancerous immortalized oral epithelial cells (OEC) and OSCC cell lines^[84,85]^. In a preliminary study by Fujiwar *et al.*^[58]^, co-culture with Fn promoted the invasion of OSCC cells by upregulating EMT genes. Another study demonstrated that Fn infection could upregulate the level of lncRNA MIR4435-2HG, which specifically binds with miR-296-5p to weaken the ability of miR-296-5p to silence its target gene Akt2, subsequently activates the expression of snail1, and eventually accelerates EMT in the infected OECs [Figure 2F]^[86]^. 

### Extracellular product in tumor microenvironment

Different researchers using a similar *in vitro* method demonstrated that Fn infection enhances migration of human CRC-derived HCT116 cells^[87]^. These include inducing secretion of IL-8 and C-X-C motif chemokine ligand 1 (CXCL1), upregulating caspase activation and recruitment domain 3 (CARD3) to activate autophagy, and activating NF-κB to regulate miR-1322 or enhancer of B cell-dependent Keratin7-antisense/Keratin7 (KRT7-AS/KRT7), which are associated with increased metastatic potential and poor prognosis^[88-91]^.

In addition, Fn-infected CRC cells release exosomes carrying metastasis-related miR-1246/92b-3p/27a-3p and CXCL16/IL-8, particularly strongly inducing metastasis. These exosomes are then internalized to induce upregulation of β-catenin, cellular MYC proto-oncogene, cyclin D1, and the mesenchymal markers in CRC cells, implying broad cancer promoting effect of Fn exosomes and driving uninfected recipient cells toward a pro-metastatic phenotype^[92]^.

As a Gram-negative bacterium, Fn can secret outer membrane vesicles (OMVs), containing proteins, lipoproteins, phospholipids, and LPS, which act as a delivery system for virulence factors [Figure 2B]^[93]^. OMVs interact with host epithelial cells through surface proteins and adhesion molecules^[94]^. Intra-OMV proteases of Fn further degrades host E-cadherin, which facilitates bacterial invasion, inflammatory responses, and EMT.

### Angiogenesis

Angiogenesis is necessary to provide nutrients and oxygen to tumor cells for further growth and tumor progression. Fn potentially activates the autocrine function of endothelial cells, resulting in a higher release of VEGF, VEGFR1, and VEGFR 2, which is favorable for proliferation and metastasis [Figure 2G]^[95]^. In another way, Fn-attracted MDSCs reduce infiltration of T cells into the tumor and increase expression and promote secretion matrix metalloproteinase 9 (MMP-9) and MMP-13 to promote angiogenesis^[79]^.

## ROLE OF Fn IN CANCER DRUG RESISTANCE

The development of drug resistance is the major cause of cancer therapy failure, affects cancer progression, and results in a poor prognosis. A growing body of evidence implies that microorganisms could modulate the host response to chemotherapeutic and immunotherapeutic drugs. Increased Fn abundance tracks with tumor stage and is associated with chemotherapy response^[90,96-99]^. Inhibiting the growth of Fn significantly augments the first-line chemotherapy efficiency in CRC^[100]^. Specific mechanisms of drug resistance may vary with different cancers and drugs. Currently, the research on Fn-mediated drug resistance mainly focuses on 5-fluorouracil (5-FU), oxaliplatin (OXA), and cisplatin (CDDP).

### Autophagy pathway

**Figure 3:**
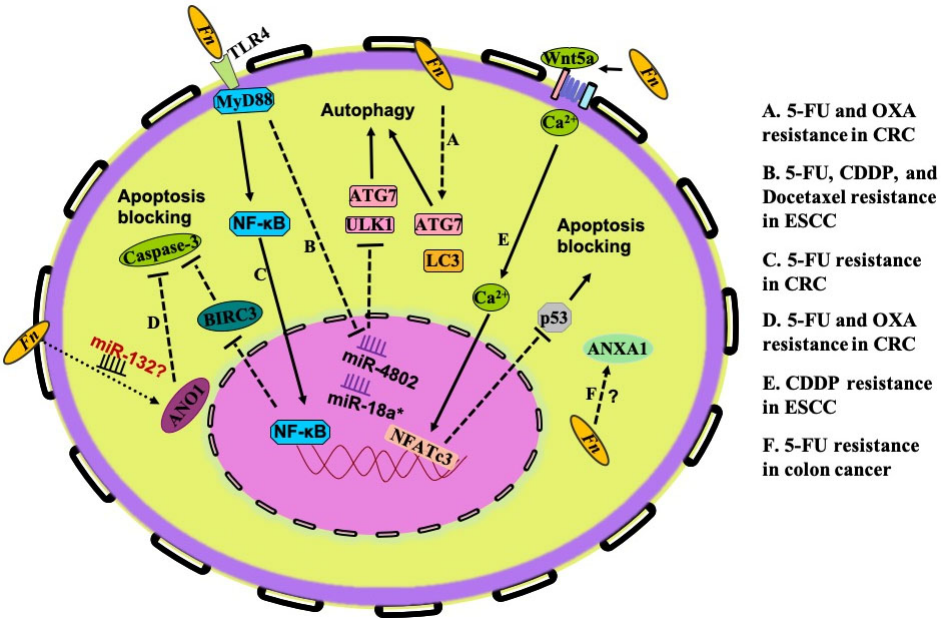
Specific mechanisms of drug resistance induced by Fn. The research on Fn-mediated drug resistance mainly focuses on autophagy activation (A,B) and apoptosis blockade (C-F). (A) Fn modulates endogenous LC3 and ATG7 expression to induce chemoresistance against 5-FU, CDDP, and docetaxel in ESCC. (B) Fn intervention induces a selective loss of miR-4802 and miR-18a*, leading to TLR4/MYD88-dependent autophagy activation and a CRC chemotherapeutic response to OXA and 5-FU. (C) Fn-mediated TLR4/MYD88/NF-κB pathway activation induces upregulation of BIRC3, which consequently cripples the level of cleaved caspase 3 and cleaved PARP caused by 5-FU. (D) In colon cancer cells, the apoptosis effects induced by OXA and 5-FU could be prevented by Fn-induced ANO1 upregulation. (E) Fn-induced overexpression of ANXA1 confers 5-FU resistance in colon cancer cells, but the specific mechanism needs further investigation. (F) Fn may downregulate p53 expression through the non-canonical Wnt/NFAT pathway to inhibit CDDP-induced apoptosis and migration in OSCC cells. Fn: *Fusobacterium nucleaum*; 5-FU: 5-fluorouracil; OXA: oxaliplatin; CRC: colorectal cancer; CDDP: cisplatin; ESCC: esophageal squamous cell carcinoma; BIRC3: baculoviral IAP repeat-containing protein 3; ANO1: Anoctamin-1; ANXA1: Annexin A1; OSCC: oral squamous cell carcinoma; PARP: poly ADP-ribose polymerase.

The* in vitro* assay results performed by two research groups provide an insight into Fn-induced chemoresistance via the autophagy pathway in CRC and esophageal squamous cell carcinoma (ESCC) cells. They found that Fn increased the level of multiple autophagy-related genes, such as those encoding ATG7, ULK1, Beclin-1, and LC3-II. One group reported that Fn modulates the endogenous LC3 and ATG7 expression to confer chemoresistance against 5-FU, CDDP, and docetaxel in ESCC [Figure 3A]^[101]^. Moreover, the chemoresistance induced by Fn was reversed by inhibiting autophagy through ATG7 knockdown. The results from Yu’s study indicate that Fn induces CRC resistance to OXA and 5-FU. Mechanistically, Fn intervention induces a selective loss of miR-4802 and miR-18a*, leading to TLR4/MYD88-dependent autophagy activation and a CRC chemotherapeutic response [Figure 3B]^[102]^. These studies provide insight into the role of Fn in the modulation of drug resistance through the regulation of autophagy in host cells.

### Apoptosis blocking

Inhibitors of apoptosis proteins (IAPs) are characterized by the presence of baculoviral IAP repeat (BIR) domains, exerting the binding and inhibition of caspases. Baculoviral IAP repeat-containing protein 3 (BIRC3), a member of the IAP family, can inhibit apoptosis by directly inhibiting the caspase cascade to promote chemoresistance in malignancies^[103-105]^. It is reported that Fn infection protects CRCs from 5-FU-mediated apoptosis. The action mechanism is that Fn-mediated TLR4/MYD88/NF-κB pathway activation induces upregulation of BIRC3, which consequently cripples the level of cleaved caspase 3 and cleaved poly ADP-ribose polymerase (PARP) caused by 5-FU [Figure 3C]^[106]^. Anoctamin-1 (ANO1), as one of the human chloride channel proteins, is frequently upregulated in different types of human cancers and is involved in AKT and MAPK signaling activation, which plays a critical role in cancer progression. Research data indicate that Fn promotes ANO1 expression in colon cancer cells and that the OXA- and 5-FU-induced apoptosis could be prevented by ANO1 [Figure 3D]^[103]^. Based on the fact that ANO1 is a target of miR-132, which has a crucial role in CRC progression, and the effect of Fn on miRNAs, some authors hypothesized that Fn prevents apoptosis in CRC via the ANO1 pathway involved in modulation of the amounts of miRNA^[107]^. Annexin A1 (ANXA1) is a calcium-dependent phospholipid-linked protein that is involved in drug resistance, has anti-inflammatory effects, and regulates cellular differentiation, proliferation, and apoptosis. Onozawa *et al.*^[108]^ reported that Fn-induced overexpression of ANXA1 confers 5-FU resistance in colon cancer cells, but the specific mechanism needs further investigation [Figure 3E]. The nuclear factor of activated T-cells (NFAT) is a downstream effector of the non-canonical WNT/Ca^2+^ signaling pathway that has been demonstrated to promote the migration of tumor cells and restrain apoptosis^[109]^. According to a study in 2021 conducted by Da *et al.*^[110]^, Fn downregulates p53 expression to inhibit CDDP-induced apoptosis and migration of OSCC cells. Probing into the mechanism, they found that Fn may downregulate p53 and E-cadherin through the non-canonical Wnt/NFAT pathway and induce drug resistance in Cal-27 and HSC-3 of CDDP [Figure 3F].

## PREVENTION STRATEGIES

The above findings not only explain the correlation between Fn abundance and the mechanisms of tumor initiation, promotion, and progression but also raise the question of whether patients with a high abundance of Fn could benefit from an Fn-directed therapy before or concomitant with chemotherapy.

Most clinical isolates of Fn are sensitive to a number of antibiotics, including metronidazole and clindamycin and some β-lactam antibiotics. In patient-derived xenograft models of CRC with Fn enrichment, treatment with metronidazole reduced Fn load and impaired cancer cell proliferation and overall tumor growth, suggesting that Fn-abundant tumors may benefit from anti-fusobacterial therapy^[72]^. However, owing to the diversity of microbiota, implementing such an antibiotic intervention would be problematic in many ways. This is because antibiotics non-selectively kill both pro- and anti-tumoral bacteria. To avoid such a problem, a gut microbiota-modulatory therapy based on phage-guided biotic-abiotic hybrid nanomaterials was described by Zheng *et al.*^[100]^. In brief, they first isolated a phage strain from human saliva that could specifically lyse Fn. Then, they encapsulated irinotecan (IRT), a first-line drug against CRC, within dextran nanoparticles (DNPs) to form IRT-loaded DNPs (IDNPs). Finally, using a bioorthogonal reaction, they covalently linked IDNPs to azide-modified phages (A-phages) to construct a phage-guided biotic-abiotic hybrid nanosystem^[100]^. *In vivo* experiments were then carried out to demonstrate that A-phages accumulated in CRC tumors and that the oral administration of the nanosystem eliminated intra-tumor Fn, which inspires future treatment strategies for tumors with Fn abundance. Alternatively, an Fn-specific narrow-spectrum antibiotic might be beneficial, but due to concerns about antibiotic resistance for both broad- and narrow-spectrum antibiotics, strategies targeting virulence or interaction receptors between Fn and host cells may be more promising^[15]^. The Fn adhesin Fap2 and TIGIT/TLR4 may be attractive targets as they promote Fn enrichment, compromise antitumor immunity, and confer chemoresistance. An Fn-directed vaccine target at FomA (an outer membrane protein expressed by Fn that functions in bacterial co-aggregation and biofilm formation) that elicits immune response has already been tested^[111]^. However, data concerning the incidence of CRC after receiving the vaccine are still lacking. Further, Brennan *et al.*^[15]^ argued that, even if the vaccination can elicit certain types of immune responses (such as human versus T cell responses), some Fn strains still escape from the immune killing effect in their intracellular phase^[112]^. Alternatively, T cell-inducing vaccines, similar to those vaccines targeting malaria and tuberculosis, might produce a preferable strategy for Fn. Microbial ecosystem replacement, using consortia of designed microorganisms or designed cocktails of human-derived isolates, may be another option to change the tumoral microbiota that potentially harbors Fn enrichment. This approach is clinically on trial with *Clostridium difficile* and might be tested in the future to exclude Fn.

## CONCLUSION

Fn is a multidimensional bacterium that engages in interactions ranging from beneficial to detrimental in nature with other microorganisms and humans. Some researchers suggested that Fn is a passenger rather than a driver in disease states^[113]^. However, from the above review, we can conclude that Fn plays an important role in the whole process of cancer initiation, disease progression, and chemotherapy resistance, supporting the notion that Fn may have a causative role in different states of cancer rather than being a consequence of cancer development and chemoresistance. Nevertheless, it is still unknown how Fn transforms from a beneficial bacterium to a harmful one. Before we consider Fn-targeted treatments, we must obtain more knowledge about the basic biology of Fn. Two important challenges within this issue are: (i) investigating how Fn strains and levels in different organs affect cancer; and (ii) understanding the mechanistic complex interactions of Fn-microbe-host within the tumor microenvironment^[6]^. Only by continuous investigation of the mutualistic and pathogenic characters of Fn will we reveal the divergent ways that can be used for diagnostic, preventive, and therapeutic purposes.

## DECLARATIONS

### Copyright

© The Author(s) 2022.
